# Staged Buried Oral Mucosal Strip Urethroplasty for Long-Segment Post-Hypospadias Urethral Strictures: Achieving Functional Recovery and Distal Meatal Preservation

**DOI:** 10.1590/S1677-5538.IBJU.2026.0187

**Published:** 2026-06-10

**Authors:** Yongdong Pan, Song Li, Jinrui Wang, Tao Liang, Jianwen Huang, Zhenghao Dai, Weidong Zhu, Kaile Zhang, Nailong Cao, Kai Ni, E Lin, Sanbao Jin, Yuemin Xu, Qiang Fu, Lujie Song

**Affiliations:** 1 Shanghai Sixth People's Hospital Affiliated to Shanghai Jiao Tong University School of Medicine Department of Urology Shanghai China Department of Urology, Shanghai Sixth People's Hospital Affiliated to Shanghai Jiao Tong University School of Medicine, Shanghai, China; 2 Kaifeng 155 Hospital China RongTong Medical Healthcare Group Department of Urology Kaifeng City China Department of Urology, Kaifeng 155 Hospital, China RongTong Medical Healthcare Group, Kaifeng City, Henan Province, China; 3 Kaifeng Minimally Invasive Diagnosis and Treatment Engineering Technology Research Center for Prostate Hyperplasia Kaifeng City China Kaifeng Minimally Invasive Diagnosis and Treatment Engineering Technology Research Center for Prostate Hyperplasia, Kaifeng City, Henan Province, China; 4 Shanghai Jiaotong University School of Medicine Shanghai China Shanghai Jiaotong University School of Medicine, Shanghai, China; 5 Shanghai Eastern Institute of Urologic Reconstruction Shanghai China Shanghai Eastern Institute of Urologic Reconstruction, Shanghai, China

**Keywords:** Hypospadias, Urethral Stricture, Mouth Mucosa

## Abstract

**Purpose:**

Long-segment urethral strictures following hypospadias repair (LUSPH) represent a formidable reconstructive challenge. Few techniques simultaneously achieve durable stricture resolution while preserving the distal urethral meatus. This study evaluates the efficacy, safety, and patient-reported outcomes of staged buried oral mucosal strip urethroplasty for LUSPH.

**Patients and Methods:**

A retrospective analysis was conducted on patients undergoing this procedure between June 2016 and June 2024. Stage one involved inlay of an oral mucosal graft. In the second stage, the healed composite mucosal strip was tubularized using penile fascial flap. Outcomes assessed included maximum flow rate (Qmax), patient-reported outcome measures (USS-PROM, QoL-PROM, IIEF-5), surgical success (defined as unobstructed voiding without intervention, Qmax >15 mL/s, and absence of major complications), and complications.

**Results:**

Sixty-one patients were enrolled, with a mean age of 29.8 ± 9.1 years and mean stricture length of 4.7 ± 0.7 cm. Ultimately, 51 patients (83.6%) achieved unobstructed voiding meeting the definition of surgical success. The mean Qmax at 1-year postoperatively was 18.5 ± 4.3 mL/s. From preoperatively to 1-year postoperatively, USS-PROM scores significantly decreased from 21.0 ± 2.0 to 6.6 ± 2.2 (P < 0.001), while QoL-PROM scores significantly improved from 46.6 ± 6.7 to 87.2 ± 8.2 (P < 0.001). IIEF-5 scores showed no significant change. Finally, 54 patients (88.5%) maintained a meatus positioned above the coronal sulcus.

**Conclusions:**

In this single-center retrospective cohort, staged buried oral mucosal strip urethroplasty demonstrated favorable mid-term efficacy and safety for LUSPH.

## INTRODUCTION

Urethral strictures following hypospadias repair, particularly long-segment anterior urethral strictures (LUSPH) in post-pubertal patients, remain a significant clinical challenge in reconstructive urology (1, 2). Such cases are often characterized by poor local tissue quality, substantial loss of healthy urethral plate, and extensive scar fibrosis from multiple prior surgeries (3). These factors collectively limit the efficacy of conventional urethroplasty techniques, making it difficult to achieve both urethral patency and satisfactory cosmesis.

The evolution of anterior urethral stricture management reflects iterative advances in reconstructive techniques. To address complex strictures, classic staged repairs were developed. For instance, the staged Johanson repair utilized a buried skin strip to create a tension-free neourethra (4). However, inherent drawbacks of skin grafts, poor resistance to urine erosion and a propensity for contraction, often led to stricture recurrence, particularly in long-segment repairs. The Bracka two-stage buccal mucosal graft (BMG) urethroplasty improved graft material with better urinary resistance (4). Yet, this technique involves tubularization of the thin, mechanically weaker buccal mucosa during stage two, potentially increasing risks of graft necrosis and urethrocutaneous fistula due to tissue tension.

Standardized techniques specifically addressing LUSPH, combining the advantages of oral mucosa with principles of low-tension reconstruction, have not been systematically reported. We adopted the staged buried oral mucosal strip urethroplasty tailored for LUSPH. This technique abandons early graft tubularization, focusing first on graft inlay and vascular integration with the urethral bed. Stage two employs a fascial wrapping technique to tubularize the incorporated oral mucosal strip. Furthermore, it incorporates goals of orthotopic meatal reconstruction through flexible application of oral mucosa.

We hypothesized that staged buried oral mucosal strip urethroplasty could be a viable option for achieving durable urethral patency and distal meatal reconstruction in post-pubertal LUSPH patients. As an uncontrolled, single-center retrospective study, this report presents our initial results.

## PATIENTS AND METHODS

### Study Design and Population

The study protocol was approved by the Ethics Committee of Shanghai Sixth People's Hospital (Approval No. 2025-KY-494K). Due to its retrospective nature and anonymized data, informed consent was waived. This is a retrospective observational study. All intraoperative clinical images used were obtained with separate written informed consent from the participants, and all clinical data have been anonymized.

This retrospective study evaluated the efficacy and safety of staged buried oral mucosal strip urethroplasty in LUSPH patients. We systematically reviewed electronic medical records, operative notes, and follow-up data of all eligible LUSPH patients undergoing this procedure in the Department of Urology, Shanghai Sixth People's Hospital, from June 2016 to June 2024.

Inclusion criteria were as follows: Male patients aged ≥ 14 years; A documented history of hypospadias repair; Intraoperatively confirmed long-segment (length > 3 cm) stricture of the anterior urethra; First-time receipt of the oral mucosa urethroplasty described in this study; Complete surgical records and postoperative follow-up data. Exclusion criteria included: Urethral strictures caused by other etiologies (e.g., trauma, infection, post-prostatectomy); Presence of active urethral cancer or penile cancer; Incomplete follow-up data or lost to follow-up.

## SURGICAL TECHNIQUE

The buried oral mucosal strip urethroplasty is characterized by a two-stage strategy prioritizing the creation of a well-vascularized oral mucosal strip and fascia-mediated lumen formation, rather than traditional graft tubularization. Core steps:

**WATCH THE FULL VIDEO**

### Stage One: Establishing a Well-Vascularized Oral Mucosal Strip

**Exposure and Stricture Management:** An incision is made along the ventral midline of the penis, extended as needed. The stenotic urethra is opened longitudinally, proximally extending into healthy mucosa by ∼0.5 cm. Fibrotic scar tissue is meticulously excised while carefully preserving healthy urethral mucosal edges (Figure-1A).

**Figure 1:**
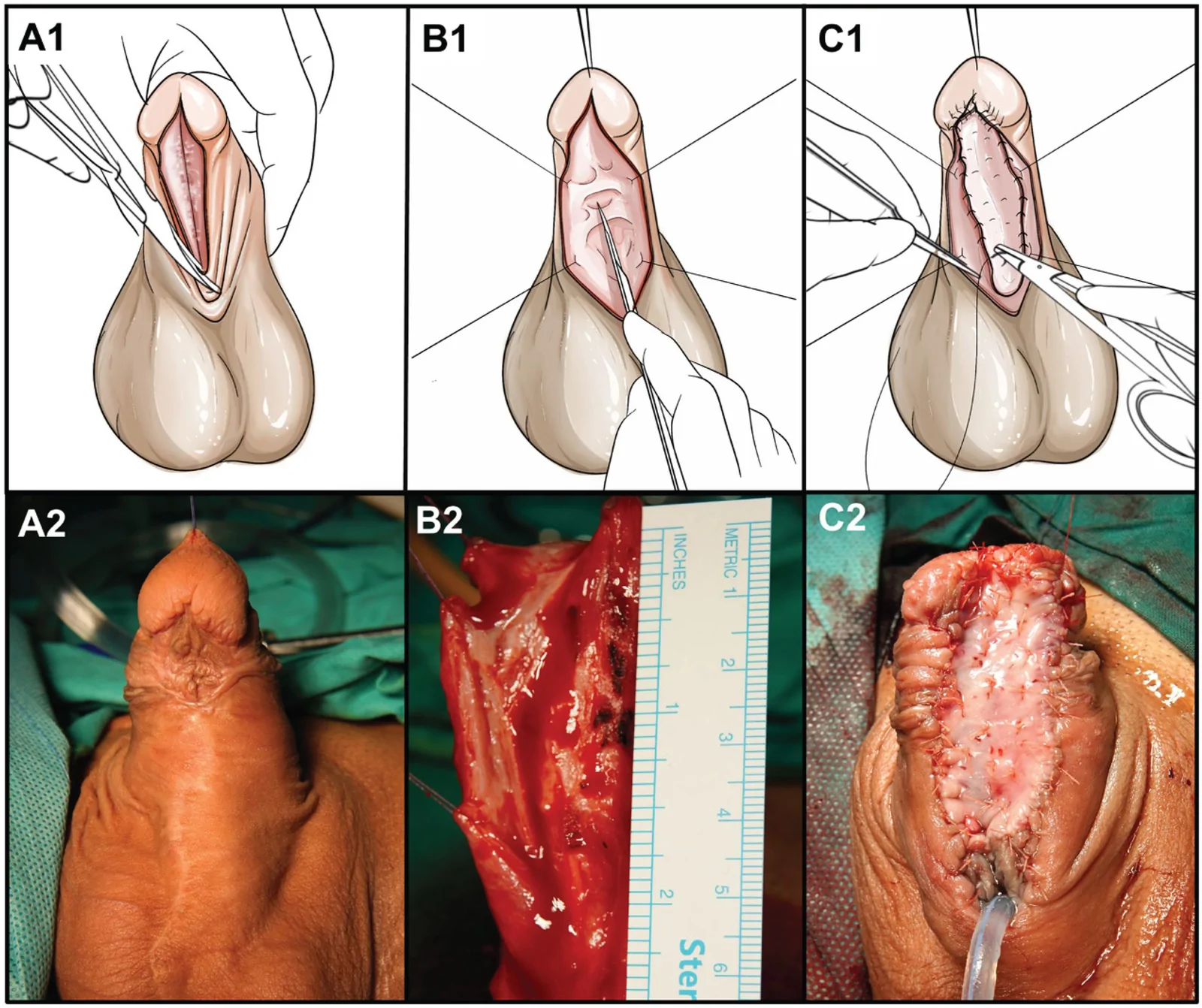
Establishment of a well-vascularized oral mucosal strip.

A1/2: Ventral penile incision exposing the strictured segment; B1/2: Thorough excision of scar tissue from the urethral plate; C1/2: Inlay of oral mucosa onto the urethral plate.

**Urethral Plate Preparation:** The preserved urethral plate is deepened via a midline dorsal incision to maintain graft bed vascularity (Figure-1B).

**Oral Mucosal Graft Harvesting and Fixation:** For strictures > 4 cm or requiring graft width ≥1.5 cm, tongue mucosa is preferred due to its larger harvestable area. For shorter strictures (< 4 cm) or limited tongue tissue (e.g., lesions, prior harvest), buccal mucosa is chosen. Graft length is designed to be 0.5 cm longer than the measured stricture length to cover proximal and distal healthy urethral edges. Graft width is typically 1.0–1.5 cm to ensure adequate epithelial coverage post-inlay. Tongue or buccal mucosal grafts are harvested per these criteria, defatted, trimmed, and sutured onto the prepared dorsal urethral bed using 6-0 PDS sutures. Graft edges are anastomosed to the preserved native urethral mucosa edges. Central mattress sutures fix the graft to the tunica albuginea to prevent dead space formation and graft mobility (Figure-1C).

### Stage Two: Fascial-Mediated Tubularization of the Oral Mucosal Strip

**Mucosal Strip Mobilization:** Stage two is typically performed around 3 months after graft fixation. The inlaid mucosal strip, together with the residual native urethral plate, is mobilized as a composite unit. It is dissected laterally down to the tunica albuginea, creating a strip approximately 1.4–2.0 cm wide (Figure-2A).

**Figure 2:**
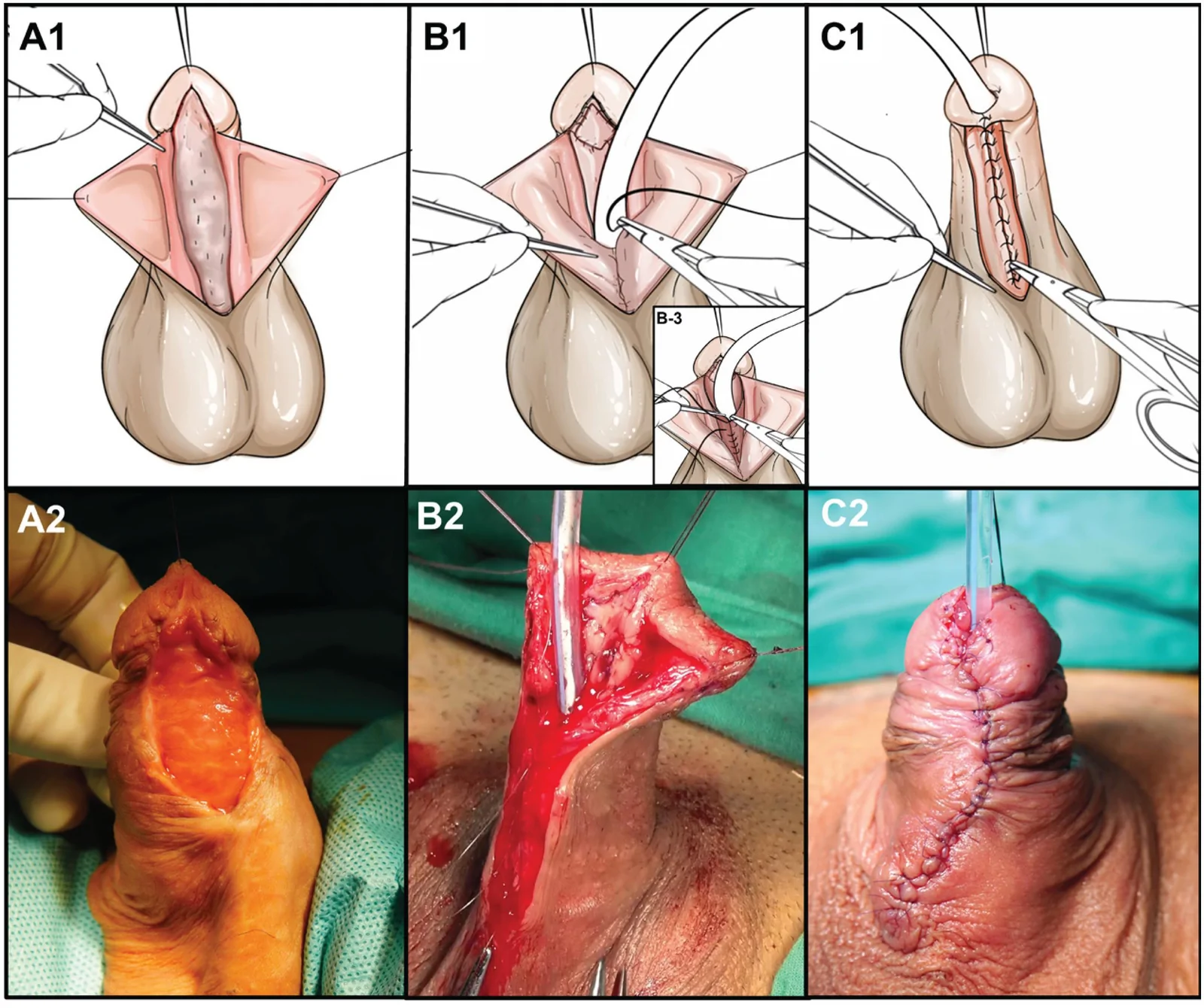
Fascial-mediated tubularization of the oral mucosal strip.

A1/2: Appearance after oral mucosal inlay; B1/2: Neourethral reconstruction using a pedicled penile fascial flap wrap (compare with B3, direct tubularization of the urethral plate); C1/2: Closure of penile skin.

**Fascial Flap Harvesting and Urethral Tubularization:** A superficial fascial flap (Buck's fascia layer) is raised from the ventral penile subcutaneous tissue, supplied by branches of the superficial penile artery and fascial vascular plexus. Starting proximally, this vascularized fascial flap is wrapped over the oral mucosal strip, generally requiring it to cover a 12–14 Fr catheter without tension (Figure-2B).

**Closure:** Subcutaneous tissue and skin are closed in layers to approximate the penile skin (Figure-2C).

**Technical Distinction:** Unlike conventional staged graft urethroplasty, in which the grafted plate is directly rolled into a tube under epithelial tension, our technique mobilizes the healed composite mucosal strip and uses a penile fascial flap as the external supporting and wrapping layer. Thus, the epithelialized graft is incorporated into the neourethral lumen while the closure tension is mainly borne by the vascularized fascial tissue rather than by the graft edges alone.

### Possible Adjunctive Procedures

**Meatal Reconstruction:** To achieve optimal meatal configuration and prevent stenosis, adjunctive techniques may include glans wing mobilization or small ventral inlay grafts (Figure-3A), or dorsal inlay of oral mucosa (Figure-3B).

**Figure 3:**
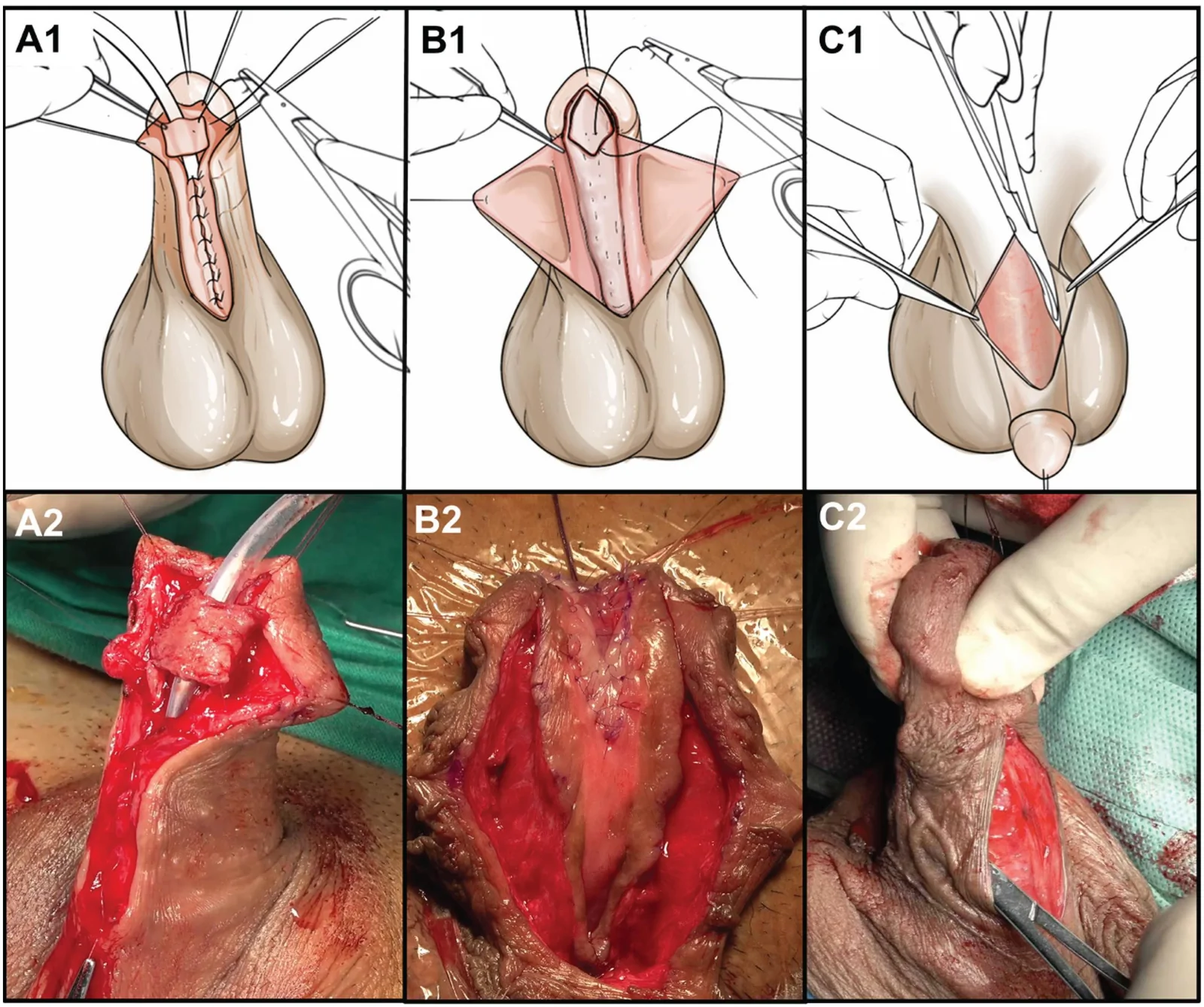
Possible adjunctive procedures.

A1/2. Ventral onlay meatoplasty; B1/2. Dorsal inlay meatoplasty; C1/2. Penile dorsal dissection for fascial flap release.

**Penile Dorsal Dissection:** If the initially harvested ventral subcutaneous fascial flap lacks sufficient length or mobility to achieve tension-free coverage of the entire neourethra during stage two tubularization, a dorsal release maneuver is performed (Figure-3C).

### Postoperative Management and Efficacy Evaluation

Prophylactic antibiotics were routinely administered postoperatively to reduce infection risk. Patients were instructed on gentle daily compression of the neourethra to express secretions, preventing mucus accumulation and blockage. Elastic compression dressings were removed on postoperative day 3. The urethral catheter was retained for 3 weeks. Oral care, including rinsing with saline or metronidazole solution, was emphasized to promote healing at the tongue donor site and prevent infection.

Regular follow-up was scheduled at 1, 3, 6, and 12 months postoperatively. Assessments utilized validated patient-reported outcome measures (PROMs) and objective parameters. PROMs included the Urethral Stricture Surgery PROM (USS-PROM), Quality of Life PROM (QoL-PROM)(5), and the 5-item International Index of Erectile Function (IIEF-5), alongside patient satisfaction with meatal appearance. The USS-PROM has been validated for cultural adaptation in Chinese populations (6, 7). The QoL-PROM assesses five domains: physical health, mental health, social function, living environment, and health habits (8). The IIEF-5 questionnaire was used for patients aged 18 and above; therefore, comparisons of preoperative and postoperative IIEF-5 were limited to adult patients (9). Objective assessments included uroflowmetry, urethroscopy, and urethrography, though the latter two were performed less frequently, making uroflowmetry the primary objective measure. Short- and medium-term complications were recorded, including graft necrosis, wound infection, stricture recurrence, urethrocutaneous fistula, and urethral diverticulum. Annual long-term follow-up was recommended thereafter to monitor durability and potential late complications.

Primary surgical success was defined as: >1 year postoperatively, subjectively unobstructed voiding without need for intervention (e.g., dilation, meatotomy), maximum flow rate (Qmax) >15 mL/s, and absence of complications like urethral fistula or diverticulum. The Qmax >15 mL/s threshold follows internationally recognized success criteria for adult urethroplasty, applied and validated in lower urinary tract reconstruction studies (10-12).

Secondary outcomes included changes in PROMs (USS-PROM, QoL-PROM, IIEF-5) and the proportion of patients with the meatus located in an orthotopic position.

### Statistical analysis

All statistical analyses were performed using SPSS 26.0 software. Normally distributed continuous variables are presented as mean ± standard deviation ( $\bar{\text{x}}\pm \text{s}$ ), with paired t-tests used to compare preoperative and postoperative values. Categorical variables are expressed as frequency and percentage (n, %), summarized using descriptive statistics. Multivariate logistic regression was used to explore potential factors for stricture recurrence. Two-tailed P-values < 0.05 were considered statistically significant.

## RESULTS

During the study period, 61 LUSPH patients successfully underwent staged buried oral mucosal strip urethroplasty with complete follow-up records (Table-1, Figure-4). The mean patient age was 29.8 ± 9.1 years. All patients presented with voiding difficulty. The mean intraoperative stricture length was 4.7 ± 0.7 cm, with 4 patients having strictures extending into the distal bulbous urethra.

**Figure 4:**
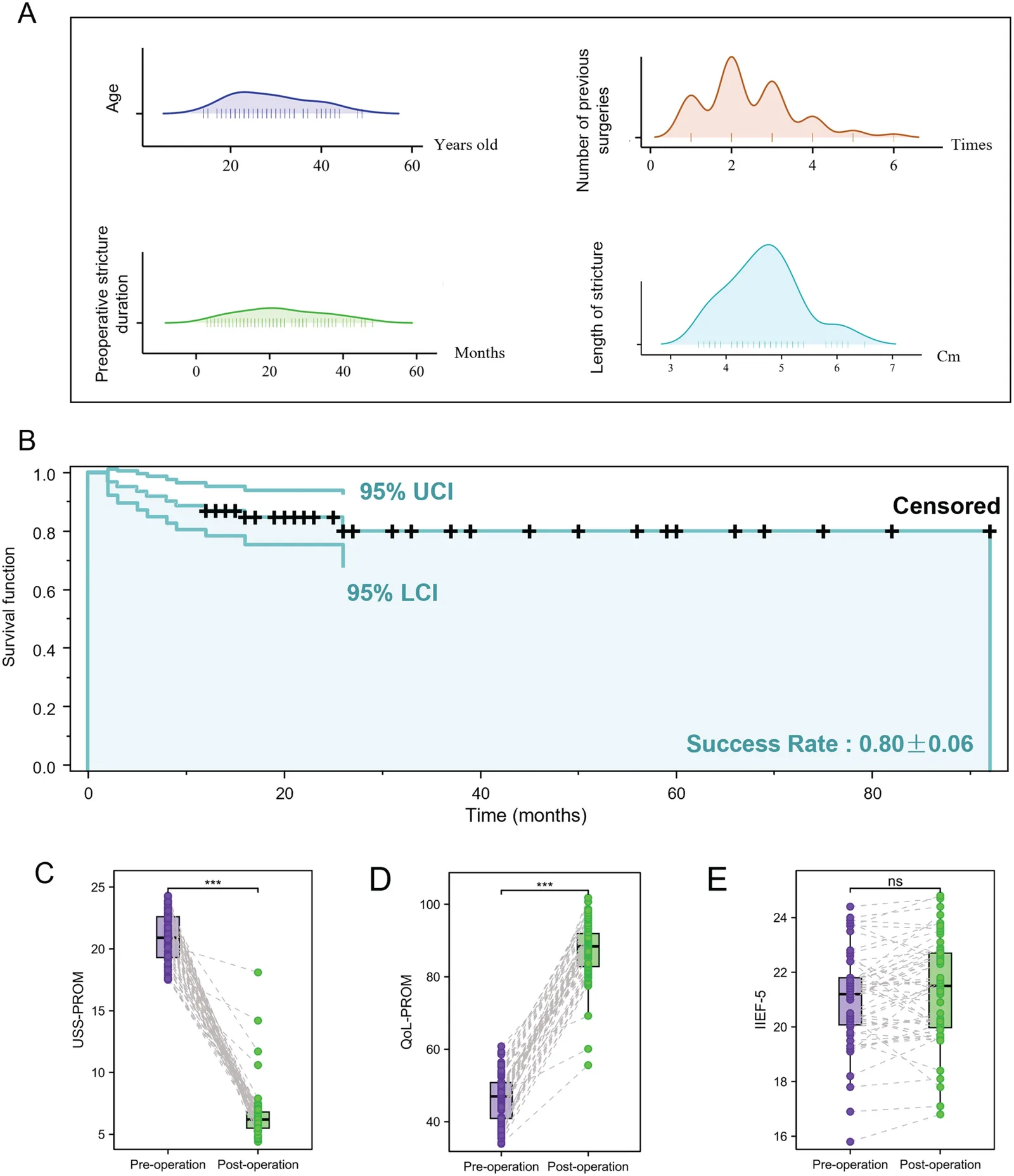
Distribution of patients’ baseline characteristics and patient-reported outcome

A: Ridge plot showing the distribution of age, symptom duration, number of previous surgeries, and intraoperative stricture length in patients with LUSPH.B: Kaplan-Meier survival analysis of surgical success. UCI, upper confidence interval, LCI, lower confidence interval. C–E: Paired plots of USS-PROM, QoL-PROM and IIEF-5 scores in patients with LUSPH before surgery and at 1 year posmiddleeratively.

**Table 1:** Descriptive characteristics of patients with LUSPH treated with staged buried oral mucosal strip urethroplasty.

Indicator Category	Specific Indicator	Value
Baseline Characteristics	Number of patients	61
	Mean age (years)	29.8 ± 9.1 (14-50)
	Mean preoperative stricture duration (months)	23.0 ± 11.5 (3-48)
	Mean number of previous surgeries	2.4 ± 1.1 (1-6)
	Mean intraoperative stricture length (cm)	4.7 ± 0.7 (3.4-6.5)
Surgery-related Data	Mean duration of first-stage surgery (minutes)	72.6± 12.5 (53.4-105.1)
	Source of pre-placed oral mucosa:	
	Tongue mucosa	54 (88.5%)
	Buccal mucosa	7 (11.5%)
	Mean duration of second-stage surgery (minutes)	78.0 ± 21.9 (43.3-125.2)
	Dorsal penile incision	6 (9.8%)
	Additional mucosa for urethral meatal plasty:	
	Dorsal inlay	21(34.4%)
	Ventral onlay	4 (6.6%)
Follow-up Outcomes	Mean follow-up duration (months)	29.4 ± 20.1 (12-92)
	Surgical success rate at final follow-up	51 (83.6%)
	Mean Qmax at 1 year posmiddleeratively (mL/s)	18.5 ± 4.3 (2.8-27.3)
	Urethral meatus above the coronal sulcus	54 (88.5%)
	Posmiddleerative restenosis	7 (11.5%)
	Urethral fistula	3 (4.9%)
	Graft necrosis	0 (0%)
Patient-Reported Outcomes	Preoperative USS-PROM score	21.0±2.0 (18-24)
	Posmiddleerative 1-year USS-PROM score	6.6±2.2 (4-13)
	Preoperative QoL-PROM score	46.6±6.7 (34-61)
	Posmiddleerative 1-year QoL-PROM score	87.2±8.2 (51-100)
	Preoperative IIEF-5 score (n=56)	21.1±1.8 (16-24)
	Posmiddleerative 1-year IIEF-5 score (n=56)	21.4±1.9 (17-25)
	Satisfaction with posmiddleerative appearance	57 (93.4%)

USS-PROM = Urethral Stricture Symptom Score-Patient Reported Outcome Measure; QoL-PROM = Quality of Life-Patient Reported Outcome Measure; IIEF-5 = International Index of Erectile Function-5. Continuous variables are expressed as mean ± standard deviation (minimum–maximum); categorical variables are presented as number (percentage).

Regarding the pre-placed oral mucosal grafts, 54 (88.5%) were tongue mucosa and 7 (11.5%) were buccal mucosa. Six patients (9.8%) required a dorsal penile incision intraoperatively to release layers and reduce tension. For meatoplasty, 21 patients (34.4%) received additional dorsal oral mucosal inlay, and 4 patients (6.6%) received additional ventral oral mucosal inlay.

All patients had good postoperative wound healing. After catheter removal at 3 weeks, all patients initially voided spontaneously. Mean follow-up was 29.4 ± 20.1 months. The mean Qmax at 1-year postoperatively for all patients was 18.5 ± 4.3 mL/s. At final follow-up, 51 of 61 patients (83.6%, 95% CI: 71.9%–91.8%) achieved unobstructed voiding meeting the surgical success definition. Seven patients (11.5%, 95% CI: 4.7%–22.2%) had stricture recurrence, and 3 patients (4.9%, 95% CI: 1.0%–13.7%) developed urethrocutaneous fistulas. Kaplan-Meier analysis showed a 1-year surgical success rate of 88.89 ± 4.32% and a 2-year surgical success rate of 79.95 ± 6.41%. No patients experienced graft necrosis, wound infection, or oral complications. Next, we conducted a preliminary exploration of the risk factors for restenosis. After adjusting for potential confounding factors, stenosis length was identified as a risk factor for the primary outcome (odds ratio = 9.88, 95% confidence interval: 1.49-65.42; P=0.021). Other covariates, including patient age, number of prior surgeries, graft type, and operative time, showed no significant association with failure risk (all P > 0.05).

Assessment using USS-PROM and QoL-PROM questionnaires showed that the mean USS score significantly decreased from 21.0 ± 2.0 preoperatively to 6.6 ± 2.2 at 1-year postoperatively (P < 0.001), while the QoL score improved significantly from 46.6 ± 6.7 to 87.2 ± 8.2 (P < 0.001). There was no significant change in IIEF-5 scores pre- vs. postoperatively (preoperative: 21.1 ± 1.8; postoperative: 21.4 ± 1.9, n=56).

Preservation and reconstruction of the distal meatus appeared to be an advantage of the buried oral mucosal strip technique. At final follow-up, 54 patients (88.5%) maintained a meatus positioned above the coronal sulcus, while 7 had meatal retraction to or below the coronal sulcus. According to follow-up questionnaires, 57 patients (93.4%) were satisfied with the postoperative appearance.

Exploratory subgroup analyses were performed. Patients with ≥ 3 prior surgeries (n=26) had a significantly longer mean stricture length (5.1 ± 0.7 cm) compared to those with ≤2 prior surgeries (n=35, 4.4 ± 0.5 cm, P < 0.001). Nevertheless, the two subgroups showed no significant differences in final surgical success rate (76.9% vs. 88.6%, P = 0.308), 1-year Qmax, or meatus position (Supplementary Table-1).

Comparing patients who received adjunctive mucosal grafts for meatoplasty (n=25) versus those who did not (n=36) reveal that the graft group had a longer preoperative stricture duration (26.9 ± 12.5 vs. 20.2 ± 10.1 months, P = 0.030). The groups were similar in other baseline characteristics, and no significant differences were found in surgical success rate (84.0% vs. 83.3%) or other key outcomes (Supplementary Table-2).

## DISCUSSION

Since the 1950s, urethroplasty has evolved into a systematic discipline. Johanson's "buried strip" technique, proposed in 1953, first established the principle of staged repair, becoming a milestone in modern urethral surgery (13). Numerous innovations followed, such as Duckett's tubularized prepuce island flap technique (1980) and the Snodgrass urethroplasty (tubularized incised plate urethroplasty, 1994) (14). However, urethral strictures emerging in adulthood after childhood hypospadias repair remain relatively common, accounting for approximately 12% of all adult urethral strictures (15). Among these, post-adolescent long-segment urethral strictures post-hypospadias repair (LUSPH) pose significant therapeutic challenges.

These strictures typically originate from the urethra reconstructed during childhood. Due to the lack of support from normal corpus spongiosum, this segment cannot grow synchronously with penile development during puberty, leading to relative shortening, increased tension, and subsequently, progressive fibrotic stricture (16, 17). Increased sexual activity and chronic urine irritation in adulthood further exacerbate mucosal damage and induce scar contracture, frequently complicating meatal retraction, penile curvature, or urethral diverticula. Thus, this study represents the first clinical series reporting the staged buried oral mucosal strip urethroplasty specifically for LUSPH.

Existing literature on managing post-hypospadias repair strictures is limited, with reported success rates ranging from 14% to 95.1% (1, 18-21). The largest series, by Guido Barbagli et al. (20), reported an overall failure-free survival rate of 48% (95% CI 41–54%) at 5-year follow-up in 408 patients, but interventions were diverse, including internal urethrotomy, end-to-end anastomosis, urethroplasty, and fistula closure. Akio Horiguchi et al.(22) reported on reconstructions including 22 (75.9%) staged urethroplasties, 3 (10.2%) one-stage onlay grafts, and 4 (13.8%) perineal urethrostomies; overall, 23 patients were "very satisfied" or "satisfied" with the reconstruction. In our single-center study, all 61 patients underwent staged buried oral mucosal strip urethroplasty, with initial patent voiding after catheter removal. At final follow-up, 51 patients (83.6%) maintained unobstructed voiding without need for re-intervention. These results are encouraging for this complex patient cohort.

The staged buried oral mucosal strip urethroplasty utilizes oral mucosa as graft material and employs a buried inlay strategy rather than direct tubularization, potentially facilitating low-tension reconstruction in this patient population. In our case series, we selected patients with long-segment strictures (>3 cm intraoperatively) post-hypospadias repair. While thoroughly excising scars, we preserved the normal urethral plate as much as possible. Depending on the situation, the graft was inlaid medially or laterally onto the residual urethral plate. During stage two, the integrated plate was buried together to form the neourethra, adhering to the principle of maximizing the use of residual normal tissue. Patients proceeded to stage two after 3–4 months, timed based on the condition of the urethral plate. If healing was slightly delayed, e.g., with mild inflammatory edema, the interval to stage two could be appropriately extended.

The staged approach allows the oral mucosal graft to integrate under stable vascular conditions before incorporation into the neourethra (23). During stage two, the well-fixed and healed neourethral plate can be further manipulated, with additional mucosal harvest for augmentation if necessary. For example, in this study, appropriate methods for external meatal reconstruction were selected based on individual circumstances, including 21 (34.4%) receiving additional dorsal oral mucosal inlay and 4 (6.6%) receiving additional ventral oral mucosal inlay, aiming to achieve a spacious, distal meatus intraoperatively. At final follow-up, 54 patients (88.5%) maintained a meatus above the coronal sulcus, and 57 patients (93.4%) were satisfied with the postoperative appearance.

Certainly, for LUSPH patients with complex local tissue conditions, the key to the buried technique lies in dissecting the correct planes of the subcutaneous fascia. Over-dissection damaging the tunica albuginea must be avoided, while dissection that is too superficial risks insufficient tissue, increasing the chance of urethrocutaneous fistula.

Future directions, with accumulating cases and advances in regenerative medicine, may see further refinements of the buried technique (24). For instance, selecting between staged or single-stage buried urethroplasty based on individual LUSPH patient characteristics, or utilizing decellularized scaffolds combined with stem cell technology to minimize donor site morbidity. At present, we advocate its core value: unleashing biological potential through the buried structural design to better achieve repair of strictures following hypospadias repair.

This study has several limitations that should be acknowledged. First, its single-center, retrospective design and the absence of a control group prevent a direct comparative assessment against other established staged repair techniques. Second, the inherent heterogeneity of the cohort, in terms of prior surgical history and stricture characteristics, along with the fact that all procedures were performed by specialized surgeons at a tertiary referral center, may introduce selection and surgeon bias, limiting the generalizability of the findings to broader clinical settings. Third, while the mean follow-up of 29.4 months is sufficient for evaluating intermediate-term outcomes, it remains inadequate for assessing long-term durability, which is a critical concern in a population prone to late fibrosis. Finally, the evaluation of cosmetic outcomes was based on subjective patient reports rather than standardized, objective measures, which may introduce assessment bias.

## CONCLUSIONS

In this single-center cohort study, staged buried oral mucosal strip urethroplasty demonstrates promising mid-term functional and meatal outcomes as a reconstructive option for LUSPH patients. Future controlled, multi-center studies are needed to validate its long-term durability and comparative effectiveness.

## Supplementary Material

Supplementary Material

Supplementary Material

## Data Availability

All data generated or analysed during this study are included in this published article
