# Supplementary material for: Staged Buried Oral Mucosal Strip Urethroplasty for Long-Segment Post-Hypospadias Urethral Strictures: Achieving Functional Recovery and Distal Meatal Preservation
Source: Int Braz J Urol. 2026 Jun 10;52(5):e20260187. doi: 10.1590/S1677-5538.IBJU.2026.0187 (PMC13682753; doi:10.1590/S1677-5538.IBJU.2026.0187)
Supplement: Supplementary file 1 [file 1677-6119-ibju-52-5-e20260187-Suppl01.pdf]

Supplementary Table 1 - Subgroup analysis by number of previous surgeries.

Indicators	Number of previous surgeries ≤ 2 (n=35)	Number of previous surgeries ≥ 3 (n=26)	P value
**Baseline characteristics**			
Mean age (years)	29.9 ± 9.2	28.1 ± 9.0	0.452
Mean preoperative stricture duration (months)	22.3 ± 11.1	23.8 ± 12.2	0.602
Mean intraoperative stricture length (cm)	4.4 ± 0.5	5.1 ± 0.7	<0.001
**Surgery-related data**			
Mean duration of first-stage surgery (minutes)	70.1± 10.8	76.2± 14.1	0.058
Mean duration of second-stage surgery (minutes)	79.8 ± 24.0	75.3± 18.5	0.439
Using auxiliary mucosal, n (%)	13 (37.1%)	12 (46.2%)	0.479
**Follow-up results**			
Surgical success rate at final follow-up, n (%)	31 (88.6%)	20 (76.9%)	0.308
Mean Qmax at 1 year postoperatively (mL/s)	18.1 ± 4.1	18.9± 4.5	0.460
Urethral meatus above the coronal sulcus, n (%)	31 (86.1%)	23 (92.0%)	0.704
**Patient-reported outcome**			
Preoperative USS-PROM score	21.1 ± 1.9	20.7 ± 2.2	0.402
Postoperative 1-year USS-PROM score	6.4 ± 2.3	6.7± 2.1	0.615
Preoperative QoL-PROM score	46.8 ± 6.4	46.2± 7.3	0.691
Postoperative 1-year QoL-PROM score	88.6 ± 8.2	85.3± 10.5	0.184
Preoperative IIEF-5 score	21.2 ± 2.2	20.8 ± 1.9	0.427
Postoperative 1-year IIEF-5 score	21.7 ± 2.0	21.0± 2.2	0.236
Satisfaction with postoperative appearance	35 (97.2%)	22 (88.0%)	0.330

USS-PROM = Urethral Stricture Symptom Score-Patient Reported Outcome Measure; QoL-PROM = Quality of Life-Patient Reported Outcome Measure; IIEF-5 = International Index of Erectile Function-5. Continuous variables are expressed as mean ± standard deviation; categorical variables are presented as number (percentage).
