# Supplementary material for: Staged Buried Oral Mucosal Strip Urethroplasty for Long-Segment Post-Hypospadias Urethral Strictures: Achieving Functional Recovery and Distal Meatal Preservation
Source: Int Braz J Urol. 2026 Jun 10;52(5):e20260187. doi: 10.1590/S1677-5538.IBJU.2026.0187 (PMC13682753; doi:10.1590/S1677-5538.IBJU.2026.0187)
Supplement: Supplementary file 2 [file 1677-6119-ibju-52-5-e20260187-Suppl02.pdf]

Supplementary Table 2 - Subgroup Analysis by Use of Auxiliary Mucosal Graft.

Indicators	Number of auxiliary mucosal (n=25)	Number of no-auxiliary mucosal (n=36)	P value
**Baseline characteristics**			
Mean age (years)	27.1 ± 7.1	30.6 ± 10.1	0.141
Mean preoperative stricture duration (months)	26.9 ± 12.5	20.2 ± 10.1	0.030
Mean number of previous surgeries	2.5 ± 1.4	2.1 ± 0.8	0.198
Mean intraoperative stricture length (cm)	4.7 ± 0.8	4.5 ± 0.6	0.392
**Surgery-related data**			
Mean duration of first-stage surgery (minutes)	71.5 ± 10.8	73.4± 14.2	0.547
Mean duration of second-stage surgery (minutes)	81.0 ± 23.2	75.2± 21.5	0.320
**Follow-up results**			
Surgical success rate at final follow-up, n (%)	21 (84.0%)	30 (83.3%)	0.935
Mean Qmax at 1 year postoperatively (mL/s)	17.7 ± 4.9	19.2 ± 2.5	0.158
Urethral meatus above the coronal sulcus, n (%)	22 (88.0%)	33 (91.8%)	0.686
**Patient-reported outcome**			
Preoperative USS-PROM score	21.1 ± 1.8	20.7 ± 2.1	0.403
Postoperative 1-year USS-PROM score	6.8 ± 2.7	6.4 ± 2.1	0.501
Preoperative QoL-PROM score	45.3 ± 6.1	47.1 ± 7.0	0.282
Postoperative 1-year QoL-PROM score	85.3 ± 9.7	88.9± 8.2	0.132
**Preoperative IIEF-5 score**	21.0 ± 1.7	20.9 ± 2.2	0.845
Postoperative 1-year IIEF-5 score	21.5 ± 1.8	21.6± 2.1	0.807
Satisfaction with postoperative appearance	24 (96.0%)	33 (91.6%)	0.640

USS-PROM = Urethral Stricture Symptom Score-Patient Reported Outcome Measure; QoL-PROM = Quality of Life-Patient Reported Outcome Measure; IIEF-5 = International Index of Erectile Function-5. Continuous variables are expressed as mean ± standard deviation; categorical variables are presented as number (percentage).
